# Leveraging Stakeholder Engagement and Virtual Environments to Develop a Strategy for Implementation of Adolescent Depression Services Integrated Within Primary Care Clinics of Mozambique

**DOI:** 10.3389/fpubh.2022.876062

**Published:** 2022-05-26

**Authors:** Kathryn L. Lovero, Palmira Fortunato dos Santos, Salma Adam, Carolina Bila, Maria Eduarda Fernandes, Bianca Kann, Teresa Rodrigues, Ana Maria Jumbe, Cristiane S. Duarte, Rinad S. Beidas, Milton L. Wainberg

**Affiliations:** ^1^Department of Sociomedical Sciences, Columbia University Mailman School of Public Health, New York, NY, United States; ^2^Department of Mental Health, Ministry of Health, Maputo, Mozambique; ^3^Department of Psychiatry, New York State Psychiatric Institute, Columbia University Vagelos College of Physicians and Surgeons, New York, NY, United States; ^4^Departments of Psychiatry, Medical Ethics and Health Policy, Medicine, University of Pennsylvania Perelman School of Medicine, Philadelphia, PN, United States; ^5^Penn Medicine Nudge Unit, University of Pennsylvania Health System, Philadelphia, PN, United States; ^6^Penn Implementation Science Center at the Leonard Davis Institute (PISCE@LDI), University of Pennsylvania, Philadelphia, PN, United States; ^7^Center for Health Incentives and Behavioral Economics, University of Pennsylvania Perelman School of Medicine, Philadelphia, PN, United States

**Keywords:** LMIC, mental health, depression, adolescent, implementation determinants, implementation strategies, community engagement

## Abstract

Psychiatric disorders are the number one cause of disability in adolescents worldwide. Yet, in low- and middle-income countries (LMIC), where 90% of adolescents reside, mental health services are extremely limited, and the majority do not have access to treatment. Integration of mental health services within primary care of LMICs has been proposed as an efficient and sustainable way to close the adolescent mental health treatment gap. However, there is limited research on how to effectively implement integrated mental health care in LMIC. In the present study, we employed Implementation Mapping to develop a multilevel strategy for integrating adolescent depression services within primary care clinics of Maputo, Mozambique. Both in-person and virtual approaches for Implementation Mapping activities were used to support an international implementation planning partnership and promote the engagement of multilevel stakeholders. We identified determinants to implementation of mental health services for adolescents in LMIC across all levels of the Consolidated Framework for Implementation Research, of which of 25% were unique to adolescent-specific services. Through a series of stakeholder workshops focused on implementation strategy selection, prioritization, and specification, we then developed an implementation plan comprising 33 unique strategies that target determinants at the intervention, patient, provider, policy, and community levels. The implementation plan developed in this study will be evaluated for delivering adolescent depression services in Mozambican primary care and may serve as a model for other low-resource settings.

## Introduction

Globally, psychiatric disorders are the largest contributor to burden of disease in adolescents (1). It is estimated that 90% of adolescents live in low- and middle-income countries (LMIC), and that 10–20% of these adolescents have one or more psychiatric disorders (2). Despite this, the majority of adolescents in LMIC do not have access to treatment (3, 4), and contextually appropriate strategies for delivering evidence-based adolescent mental health care are needed to expand services to these areas.

Integrating evidence-based practices for managing adolescent psychiatric disorders within primary care clinics (PCC) has been demonstrated effective in high-income countries (5) and proposed as an efficient and sustainable way to close the adolescent mental health treatment gap worldwide (4, 6). However, very limited data exist on how to effectively implement integrated mental health care in PCC settings of LMIC (4, 7). In particular, though common implementation determinants for integrated adult mental health care in LMIC have begun to emerge (8), little is known about implementation determinants for adolescent mental health care. Moreover, which implementation strategy or combination of strategies can most effectively address these determinants remains largely unstudied, especially with regard to youth mental health services (9).

Mozambique, a Lusophone country in southeastern Africa, has a population of almost 31 million, of whom nearly one-third are adolescents ages 10–24. Like other LMIC, Mozambique has an extreme shortage of mental health specialists—there are around 1.7 for every 100,000 Mozambicans, over 30 times less than in high income countries (10, 11)—and task-shared solutions are required to meet the need for mental health services. To address the adolescent mental health treatment gap, we (policymakers and mental health specialists at the Department of Mental Health of the Mozambican Ministry of Health and implementation science and mental health researchers from the United States) have formed a partnership to apply principles of implementation science to grow adolescent mental health services within the Mozambican National Health System.

Given that depression is estimated to be the leading cause of psychiatric disorder-associated disability in Mozambican youth, similar to other LMIC (1, 12), we chose to first focus on integrating screening and treatment for depression into PCC. We selected the Patient Health Questionnaire for Adolescents (PHQ-A) as the screening tool to be implemented, as it is a brief measure that can be administered by non-specialist providers and has been previously validated for identification of depression in adolescents as well as adults in Mozambique (13, 14). We selected Group Interpersonal Therapy for Adolescents (IPT-AG) (15) as the intervention to be implemented following a review of the evidence base and evaluation of the intervention fit relative to the context. Specifically, a recent meta-analysis of psychotherapies for depression in children and adolescents indicated that only IPT-A and Cognitive Behavioral Therapy (CBT) were more effective than control conditions (16), and IPT-AG has been shown effective for treatment of adolescent depression by non-specialist workers in sub-Saharan Africa (17, 18). Contextually, IPT-AG was determined to be the best fit owing to the cultural relevance of therapy content (focus on interpersonal problems and collaborative solutions). We chose primary care clinics in Maputo City, the capital of Mozambique, as sites for pilot implementation because each clinic has a mental health specialist on site that would be able to manage adverse events in this initial research phase with a highly vulnerable population. While not representative of all cultures and contexts across the country, we believed that this population would allow for determination of a core set of strategies to comprise an implementation plan that could be adapted for scale-up across diverse regions of the country.

Implementation Mapping is a five step, systematic process for developing strategies that promote the adoption, implementation, and sustainability of evidence-based interventions (19). Here, we describe the use of Implementation Mapping to design a multilevel strategy for implementing screening, referral, and treatment for depression in adolescents integrated within PCC of Maputo Mozambique. Specifically, we used virtual and in-person approaches to identify adopters and implementers, conduct a qualitative investigation of implementation determinants, and engage stakeholders to select and specify implementation strategies that comprise the finalized implementation plan.

## Materials and Methods

All study activities (Supplementary Figure 1) were conducted in Maputo, the capital city of Mozambique. The Mozambican National Health System is led by the Ministry of Health and is where the vast majority of Mozambicans receive health care. The system is organized into community-level PCC, district-level hospitals, and province-level tertiary care hospitals as well as two specialized (quaternary care) psychiatric hospitals in the Maputo and Nampula provinces. The Department of Mental Health at the Mozambican Ministry of Health is the responsible for coordinating mental health services at all levels across the country through the National Mental Health Program. Current mental health specialists include 24 psychiatrists located in tertiary and quaternary care of four provinces and around 500 psychologists (e.g., clinical, educational, organizational), 30 occupational therapists, and 550 Psychiatric Technicians spread across primary through quaternary services throughout the country (20).

All study materials and procedures were approved by the New York State Psychiatric Institute Institutional Review Board and the Eduardo Mondlane University Institutional Health Bioethics Council.

### Implementation Needs and Assets Assessment

The implementation planners comprised the authors of this article, who are implementation science and mental researchers from Columbia University as well as policymakers and mental health specialists at the Department of Mental Health of the Mozambican Ministry of Health. We represent junior, mid-level, and senior professionals in our fields, all with previous experience in mixed-methods implementation science and mental health research. We are approximately half Mozambican (*n* = 6) and half non-Mozambican (*n* = 5); all but one implementation planner is fluent in Portuguese. Our educational backgrounds range from licensed mental health professionals to doctoral level researchers and practitioners. All but two implementation planners are also mental health practitioners.

Through a series of four virtual meetings among implementation planners, we identified adopters responsible for adolescent and mental health programming at both the national level (Ministry of Health Departments of Mental Health, School and Youth Health, and Primary Health Care) and local level (Maputo City Municipal Administration Offices of Mental Health and School and Youth Health). To identify implementers, we held two in-person workshops with 14 Mozambican stakeholders to map adolescent care pathways within PCC. Selected stakeholders included mental health specialists as well as municipal, provincial, and national coordinators of mental health services across primary through quaternary levels and coordinators of PCC-level adolescent friendly health services. With the mapped care pathways, we determined all potential points of entry, referral processes, and services provided for adolescents across primary care departments and provider-types (e.g., general medicine technician, maternal and child health nurse, physician, etc.). We then used these pathways to identify potential implementers of screening (i.e., providers that serve as points of entry for primary care services) and treatment (i.e., select providers who would be trained to deliver IPT- AG).

### Identification of Implementation Outcomes and Determinants

Over an additional series of virtual meetings among planners, we selected implementation outcomes guided by Proctor's Implementation Outcomes Framework (21) and identified project-specific performance objectives for each of these based on Ministry of Health goals. We then conducted a qualitative assessment of implementation determinants with our identified adopters and implementers: key informant interviews with national and local health officials involved in adolescent (*N* = 4) and mental health programming (*N* = 4) as well as focus groups with mental health specialists (*N* = 9) and primary care providers (*n* = 3 general medicine technicians, n = 3 sexual and reproductive health counselors, *n* = 5 nurses, *n* = 1 physician) from four PCC. The four PCC included two urban clinics and two peri-urban clinics, the former characterized by providing a wider variety of services, serving a higher patient volume, and having a larger staff than the latter. Mozambican members of the implementation planners conducted four focus groups, one at each PCC. Trained research assistants (not affiliated with the Ministry of Health or primary care system) conducted key informant interviews. The first five interviews were conducted in a private room at the Ministry of Health; owing to COVID-19 related restrictions on in-person activities that occurred during data collection, the remaining three interviews were conducted over Zoom. Each interview lasted ~1 h and each focus group ~90 min. Interviews and focus groups were digitally audio recorded and written notes were taken to summarize responses, record non-verbal communication, and note any disturbances or abnormalities during the session.

Interview and focus group guides explored implementation determinants based on the Consolidated Framework for Implementation Research (CFIR) domains (22). Mozambican implementation planners transcribed all interviews and focus groups in pairs, including one person who conducted the interview and one person who was not present. Transcripts were uploaded to Dedoose for coding. Mozambican implementation planners coded all transcripts in pairs, including one person who conducted the interview/focus group and one person who was not present. All transcripts were double coded by two pairs and discrepancies resolved via consensus with the Principal Investigator and the coding pairs. Initially, qualitative data was analyzed using the best fit framework approach (8, 23), in which transcripts were coded using the CFIR constructs as a priori codes and additional emergent codes created for concepts not in the CFIR. However, following attempted coding of two focus groups and two interviews using this method, the team chose to revisit the strategy because CFIR constructs were not well fit to the data. Specifically, the existing constructs did not capture many of the contextual determinants identified in the data. Therefore, the decision was made to instead use an open-coding approach, in which transcripts were coded in full and iteratively relabeled/subcoded as needed. Each code was then summarized and examined for patterns, triangulating results based on different participant (e.g., mental health specialists vs. non-specialist, provider vs. policymaker) perspectives and data type (interviews vs, focus groups), which yielded themes related to implementation determinants. Over a series of virtual meetings among implementation planners, themes were then organized within the five CFIR domains via consensus using Miro, an online visualization and collaboration platform. Peer debriefing was used to promote validity of both methodology and interpretation; prior to data analysis, methodology was presented to and discussed with experienced implementation scientists and global mental health researchers (N = 6) not involved in the present study and, following data analysis, methods and findings were presented to and discussed with implementation scientists with (*N* = 6) and without (*N* = 4) specialization in global mental health. We conducted member checking of results with stakeholders across a series of workshops (detailed below in Selection of Implementation Strategies).

### Selection of Implementation Strategies

We held three, day-long workshops with stakeholders to review previously identified service mapping and implementation determinant data and to select, prioritize, and specify implementation strategies. Prior to workshops, the implementation planners created simplified implementation research logic models (24) for (1) the implementation process, (2) depression screening, (3) referral for depressed adolescents, and (4) treatment with IPT-AG (Supplementary Figure 2). We selected potential implementation strategies to include in logic models by first reviewing the Expert Recommendations for Implementing Change (ERIC) (25) and then tailoring strategies to the setting and program objectives or identifying new strategies for determinants not able to be targeted by existing ERIC strategies. Logic models were developed in Miro during virtual meetings among implementation planners.

Workshop participants (*n* = 15) included policymakers (from the Ministry of Health Departments of Mental Health, School and Youth Health, and Primary Health Care, the Ministry of Education and the Office of the State Secretary for Youth), providers (mental health specialists and primary care providers for adolescents from two PCC not included in previous qualitative investigation of implementation determinants), and four local, non-governmental organizations (NGOs) with experience implementing adolescent health services in PCC. The first workshop focused on the implementation process and depression screening, the second on referral and treatment, and the third on strategy specification and finalization of the implementation plan. All workshops included a mix of presentation by the implementation planners and small group interactive discussions with participants and implementation planners. Presentations by implementation planners were used to describe objectives of the project, goals of the workshops, logic models, and implementation strategy specification. Small group discussions were used to (1) elicit feedback on implementation determinants identified and strategies proposed by the implementation planners; (2) identify additional implementation strategies not initially suggested by implementation planners; (3) prioritize strategies by importance and feasibility, by placing post-its of each strategy on a 2x2 table (Supplementary Figure 3); and (4) specify strategies selected for inclusion in the final implementation plan according to Proctor's implementation strategy specification recommendations (26). Across workshops, each small group included at least one implementation planner to guide discussion, one policymaker, two PCC providers (one mental health specialist, one primary care), and one NGO representative. Temporality of implementation strategies was specified using the EPIS framework (27).

### Production of Implementation Protocols and Materials and Evaluation of Implementation Outcomes

Beginning in 2022, we will conduct a cluster randomized trial at PCC in Maputo, Mozambique. We will use mixed methods to compare the implementation outcomes selected in Task 2 (acceptability, appropriateness, penetration, retention, fidelity, sustainability) as well as patient outcomes (change in depression symptoms) in PCC implementing depression screening and IPT-AG compared to clinics continuing with care as usual. Additionally, because data around effective implementation strategies are so limited for LMIC (9), and data on mechanisms of implementation strategy effectiveness are limited in all contexts (28), we will use qualitative evaluation with policymakers, providers, adolescents, and their caregivers to explore mechanisms of implementation strategy action and effectiveness.

## Results

### Definition of Potential Implementers

Through service mapping activities, we identified potential primary care providers to screen, refer, and treat adolescents with depression. While most PCC in Mozambique have adolescent-friendly health services, they are sometimes a separate department and sometimes integrated across multiple departments (i.e., providers in various departments trained in adolescent-friendly care). Additionally, even in clinics where there is a distinct adolescent-friendly health service department, adolescents can access care through multiple entry points at PCC. Moreover, some adolescents go directly to the mental health department when seeking specialist services. Therefore, we determined all general health and mental health providers at PCC should be considered as potential implementers of adolescent depression screening. Existing referral processes varied by provider, department, and PCC. In some cases, a mental health specialist was called to the department where an adolescent was identified in need of mental health services. In others, the adolescent was given a paper referral sheet to schedule a visit with mental health services or the adolescent was verbally informed they could seek mental health services in another area of the clinic but not given a paper referral. Therefore, we determined that all PCC providers who delivered screening should be implementers of a standardized referral protocol for depressed adolescents. Finally, some, but not all, PCC in Mozambique have a co-located mental health specialist, and these co-located mental health specialists already serve a large patient population. Thus, it was determined that we should consider mental health specialists as well as non-specialists as potential implementers of IPT-AG.

### Identification of Implementation Outcomes and Determinants

Table 1 outlines the implementation outcomes and performance objectives developed by implementation planners. All outcomes but two are measured using routinely collected, quantitative clinical data. Fidelity to IPT-AG is evaluated using a checklist completed by IPT-AG supervisors during group observation. We chose to evaluate acceptability outcomes using qualitative methods so that an in-depth understanding of the factors influencing acceptability at the provider, patient, and caregiver level could be explored and applied to strategy improvement in future implementation efforts.

**Table 1:** Implementation outcomes and performance objectives for integrated adolescent depression services in Mozambican primary care.

**Outcome**	**Measure**	**Performance objective**
Acceptability	Qualitative interviews	Acceptable to providers, caregivers, & adolescents
Adoption	% PCC providers screening, referring, & delivering IPT-AG	100% screening, referral, treatment
Fidelity	% correctly completed screens; % correctly completed referrals; IPT-AG fidelity checklist score	90%, 90%, 90%
Penetration	% adolescents at PCC screened, % referred adolescents entering treatment	90%, 90%
Retention	% IPT-AG sessions completed	80%
Sustainability	Post-trial penetration & retention	90% penetration, 90% retention

*PCC, Primary Care Clinic; IPT-AG, Group Interpersonal Therapy for Adolescents*.

Analysis of qualitative data from policymakers and providers revealed barriers and facilitators to desired implementation outcomes across all CFIR domains (Table 2). Regarding intervention characteristics, we found that providers and policymakers highly valued evidence-based interventions and preferred the group format, as it allows for treatment of multiple adolescents at once and provides an opportunity for adolescents to share experiences with peers. However, there was concern that the content of IPT-AG would not be relevant to local adolescents and the need for adaptation to the context was emphasized. In IPT-AG, three sessions take place outside the group with just the provider, caregiver, and adolescent (one prior, one in the middle, and one at the end of group sessions). While involvement of caregivers in IPT-AG was considered helpful for adolescents' symptom improvement and treatment engagement, it was also viewed as a barrier because caregivers were likely to lack the funds, time, and interest to participate in therapy sessions. Moreover, a lack of support or negative relationship with the caregiver was considered common in adolescents with mental health problems thus creating a challenge in identifying an appropriate person to participate in IPT-AG sessions. Finally, the length and number of IPT-AG sessions was perceived to be a barrier, as the cost of travel to the PCC and time commitment was considered challenging for adolescents, caregivers, and providers alike who are accustomed to brief, objective interventions (e.g., medication for infectious diseases).

**Table 2:** Implementation determinants for integrated adolescent depression services in Mozambican primary care.

**CFIR Domain**	**Implementation barriers [-] and facilitators (+)**	
Intervention characteristics	+ High valuation of evidence-based interventions + Group intervention preferred ± Involvement of caregivers considered important but challenging to realize - Concern around contextual relevance of a non-locally developed intervention - Need for multiple, lengthy sessions	
Outer setting	+ Strong, intersectoral political will - Lack of existing policy and financial resources - Low MH literacy and high stigma at the community-level	
Inner setting	± Specialized health services for adolescents, but with limited personnel/space/privacy - Lack of incentive to prioritize MH - Lack of communication between PCC departments about services available - Lack of coordination between PCC services and poor referral systems - Frequent provider turnover	
Individual characteristics	**Patients**	**Providers**
	+ Depression recognized as common problems among adolescents + Caregivers motivated to seek help when MH interferes with school and home life - Adolescents have difficulty identifying or describing their own mental health problems - Caregivers more likely to seek help for an externalizing disorder/substance use than internalizing disorder - Caregivers often don't accompany adolescent at PCC	+ Motivated to improve MH - Limited confidence in being able to deliver MH services - Lack of MH knowledge and MH stigma
Process	**Preparation phase**	**Implementation phase**
	+ Engagement with administrators & all PCC services + Engagement between MH and other departments at the Ministry of Health + Elaboration of a clearly structured implementation plan - Lack of engagement between implementation planners and community stakeholders	+ Ongoing supervision, monitoring, and technical support after training - Lack of ongoing engagement between implementation planners and local stakeholders

^+^*Implementation Facilitator; ^−^Implementation Barrier; MH, Mental Health; PCC, Primary Care Clinic*.

At the level of the outer setting, adolescent mental health was considered a policy priority across multiple health sectors. However, extant funding and policy for adolescent mental health was extremely limited. Moreover, participants described community mental health literacy as low and stigma as high, citing a common cultural belief that mental health problems are a moral failing, spiritual deficit, or a normal part of adolescence and not a medical condition that, in turn, contributes to limited care-seeking and adherence. At the level of the inner setting, participants highlighted the existence of adolescent-friendly health services at PCCs as an implementation facilitator, but indicated that these services have limited personnel, space, and privacy. Additional barriers of the inner setting included a lack of incentive to prioritize mental health among other health needs, limited communication between PCC departments and a corresponding lack of awareness of services offered at each, a lack of coordination between PCC services and poor referral systems that result in long wait times and loss of patients, and frequent provider turnover at the PCC.

Implementation determinants at the level of the individual were grouped into those regarding providers and those regarding patients, including both adolescents and their caregivers. PCC providers were highly motivated to address adolescent mental health, though non-specialists felt they had limited mental health knowledge and were unsure they would be capable of providing mental health services. Despite community-level stigma regarding mental health and a general lack of knowledge around treatment of mental health problems, participants shared that depression and anxiety were perceived as common, and therefore less stigmatized, problems among adolescents themselves. Still, there was concern that adolescents have difficulty identifying or describing their own mental health problems. Additionally, participants described caregivers as motivated to seek treatment when their adolescent was having problems at home or in school, whether or not they were able to name the source as a mental health problem. However, caregivers were also described as having limited involvement in or knowledge of their adolescent's emotional wellbeing and described as less likely to seek help for an internalizing disorder, such as depression or anxiety, than for an externalizing disorder or substance use. Moreover, adolescents most often are not accompanied by a caregiver at their PCC visits.

Finally, at the implementation process-level, participants used their experiences with previous health program implementation efforts to reflect on potential determinants of implementing adolescent depression services in PCC. Engagement between implementation planners and PCC administrators as well as all PCC services and engagement between the Mental Health Department and other departments at the Ministry of Health were considered major facilitators for implementation preparation, as was clear elaboration of program objectives, roles, activities, timelines, budget and expected outcomes. Lack of engagement between implementation planners and community stakeholders was cited as a critical barrier to preparation. In the implementation phase, lack of ongoing engagement between implementation planners and stakeholders at the local political, PCC, and community levels was perceived to be a barrier, whereas ongoing supervision of providers, monitoring of implementation, and technical support was a facilitator.

### Implementation Strategy Selection

We developed 42 potential strategies to target implementation determinants (Table 3). We then created simplified logic models to present and discuss with workshop participants (Supplementary Figure 2).

**Table 3:** Implementation strategies and their prioritization for integrated adolescent depression services in Mozambican primary care.

**Strategy type**		**Strategy**	**Priority**
Implementation process	How to prepare	Create detailed implementation plan	1
		Share implementation plan with national and local policymakers	1
		Obtain approval and commitment from PCC directors	1
		Create intervention team including implementers and adopters at PCCs	1
		Collaborate with intervention team to create intervention flowchart	1
		Identify person at PCC to serve as intervention team lead	1
		Conduct community awareness activities with Ministry of Health and Ministry of Education	1
		Conduct awareness presentations at PCC	1
		Base training in real cases	1
		Supervise IPT-AG providers	1
	How to monitor	Create a screening record	1
		Meetings between intervention team lead and implementation planners	1
		Continuous communication between implementation planners and team lead	1
		Meetings with implementation planners and intervention team	1
		Conduct refreshment training for screening and IPT-AG providers	1
Depression screening	Who/when/where	Screening in the waiting room prior to consult	2
		Screening self-completed in the waiting room	2
		Support in self-completion by administrative personnel	4
		Screening by all PCC providers	1
		Screening by all adolescent-friendly PCC providers	1
	How to deliver	Distribute support materials for screening	1
		Use non-stigmatizing language to introduce screen to adolescents	1
		Identify adequate space for screening	1
		Use a digital screen that auto-calculates scores	2
Referral to treatment	How to deliver	Use non-stigmatizing language to give feedback on screen results	1
		Provide psychoeducation following positive screen	1
		Bring adolescent with positive screen directly to MH department	1
		Provide initial IPT-AG session on day of screening	2
		Identify caregiver to participate in IPT-AG sessions with adolescent	1
		Call adolescent and/or caregiver on day prior to initial IPT-AG session	1
Depression treatment	Who/when/where	Training of at least 3 providers in each PCC	1
		MH specialist and general provider deliver groups together	1
		Creation of morning and afternoon groups	2
		Creation of Saturday groups	1
		Weekly group sessions	1
		Biweekly group sessions	2
		Identify adequate space for sessions	1
	How to deliver	Educate adolescent about IPT-AG	1
		IPT-AG provider guided by tablet	2
		Age-appropriate group composition	1
		Call adolescent and/or caregiver on day prior to each session	1
		Include caregivers remotely when they are unable to join session at PCC	1

*PCC, Primary Care Clinic, IPT-AG, Group Interpersonal Therapy for Adolescents, MH, Mental Health*.

Among the additional implementation strategies suggested by workshop participants, all were captured in the existing strategies proposed by the implementation planners (i.e., a more detailed strategy encompassed within a proposed strategy or a broader strategy that encompassed multiple proposed strategies). Therefore, just the initial 42 potential strategies were ranked by importance and feasibility. We quantified prioritization numerically where 1 = important and feasible, 2 = important but not feasible, 3 = feasible but not important, and 4 = not important nor feasible (Table 3).

All but eight (19.0%) strategies were determined to be both important and feasible. Conducting depression screening in the waiting room prior to the consultation was considered important, as it would minimize burden on the provider, but was thought to be unfeasible owing to the lack of privacy in the waiting room and available personnel who would be capable of administering the screen. Having the adolescent self-complete the screen in the waiting room was considered important, again because of minimization of provider burden, but unfeasible owing to adolescents limited literacy, mental health awareness, and previous experience indicating adolescents are less likely to respond to screens accurately without a provider's assistance. Having administrative personnel assist the adolescent in screen completion was considered both unimportant and unfeasible, as participants did not feel these personal would have the time nor the capability to help adolescents complete screens more accurately. Finally, use of a digitized screen by providers was considered important as its auto-calculation of scores reduces administration time, promotes fidelity, and allows for remote quality assurance, but was thought to be unfeasible because providers do not use electronic systems for any other services and thus may encounter challenges maintaining a device solely for screening purposes (e.g., inconsistent access to a power source at the PCC to charge the clinic, competition or resentment from providers who do not screen and thus are not given a mobile device).

Regarding referral, the strategy of providing the first IPT-AG session on the day of positive screen was considered important, as it would promote adolescents' entry into mental health care, but also unfeasible, because it is unlikely that treatment providers would have time without advanced notice and, more significantly, because the first IPT-AG session is meant to occur with the adolescent and their caregiver, but adolescents are commonly unaccompanied by a caregiver at primary care visits. Regarding treatment, weekly sessions were considered important and feasible while biweekly sessions were considered important but not feasible; biweekly sessions were not thought to increase the likelihood an adolescent would be able to attend and would also make the length of treatment twice as long, which participants indicated would hinder adherence over time. Moreover, offering morning and afternoon groups was ranked as important, because some Mozambican adolescents attend school in the morning and some in the afternoon, but infeasible, as it would be difficult for a single treatment provider to fit groups at both times in their patient load. Finally, having the IPT-AG provider guided by a tablet during treatment facilitation was considered important, as it would increase fidelity and allow remote quality monitoring, though participants believed this to be unfeasible for the same reasons as having a digitized screen.

Of the eight strategies not considered both important and feasible, seven were not included in the final implementation plan and one was collapsed within another strategy. Since morning and afternoon groups as well as Saturday groups were considered important to offer, but multiple group times was considered infeasible for providers, we combined them into one strategy “Creation of morning, afternoon, and Saturday groups” based on the availability of both adolescents and providers. Additionally, we initially proposed 1) all PCC providers and 2) all adolescent-friendly service providers as two different strategies for screening implementers. Since both strategies were deemed feasible and important, and adolescent friendly-service providers are a type of PCC provider, we combined the two strategies and used the inclusive terminology, all PCC providers, to name the strategy in the final plan. Therefore, in the final implementation plan, we included a total of 33 distinct implementation strategies.

In the final workshop, participants worked with implementation planners to specify all 33 strategies, including the actor, action, target, temporality, and dose. We then completed the strategy specification by adding in the ERIC strategy match, the strategy outcomes targeted, and the justification for inclusion of the strategy (Table 4). Our implementation strategies spanned 20 distinct ERIC strategies, with the most common being “revise professional roles” (*n* = 5 selected strategies) and “intervene to promote uptake and adherence” (*n* = 4 selected strategies). Two of the 33 strategies, “use non-stigmatizing language to introduce the screen” and “use non-stigmatizing language to discuss screen results” were not derived from ERIC strategies and we were unable to identify an appropriate corresponding ERIC strategy in *post-hoc* comparison.

**Table 4:** Implementation strategy specification for integrated adolescent depression services in Mozambican primary care.

**ERIC match**	**Adapted strategy definition**	**Actor**	**Action**	**Target**	**Temp**.	**Dose**	**Outcomes affected**	Justification^*^
**Implementation process strategies**								
Develop formal implementation blueprint	Create detailed implementation plan	IP	Develop document of project objectives, roles, activities, timeline, budget, and expected outcomes	I, A	Prep	Once	Adoption, sustainability	Elaboration of a clearly structured implementation plan; Lack of engagement between implementation planners and community stakeholders
Involve executive boards	Share implementation plan with national and local policymakers	IP	Present and deliver physical copy of implementation plan to Ministry of Health, Ministry of Education, National/Provincial/District Health Departments	A	Prep	Once	Adoption, sustainability	Engagement between MH and other departments at the Ministry of Health; Lack of engagement between implementation planners and community stakeholders
Obtain formal commitments	Obtain approval and commitment from PCC directors	IP	Present and request formal (signed) authorization of implementation plan to PCC administration	A	Prep	Once	Adoption, sustainability	Engagement with administrators & all PCC; Lack of engagement between implementation planners and community stakeholders
Organize clinical implementation team meetings	Create intervention team including implementers and adopters at PCCs	IP	Form intervention team at each PCC including all screening and treatment providers	I	Prep	Once	Acceptability, adoption, sustainability	Lack of coordination between PCC services and poor referral systems
	Collaborate with intervention team to create intervention flowchart	IP, I	Hold workshop to elaborate PCC-specific logistical details of screening (e.g., location), referrals (e.g., who completes warm hand-off to MH department), and treatment (e.g., who makes pre-session reminder calls)	I	Prep	Once	Acceptability, adoption, fidelity	Lack of coordination between PCC services and poor referral systems
Identify and prepare champions	Identify person at PCC to serve as intervention team lead	IP, A	Work with PCC administration to select one implementer with characteristics of leadership, flexibility, and self-motivation	I	Prep	Once	Adoption, fidelity	Lack of coordination between PCC services and poor referral systems
Increase demand	Conduct community awareness activities with Ministries of Health and Education	IP	Develop materials (e.g., presentations, flyers) for MH literacy, stigma reduction, and program promotion to be delivered in schools and by community health workers	C	Prep	Cont.	Acceptability, penetration	Low MH literacy and high stigma at the community-level; Lack of engagement between implementation planners and community stakeholders
Conduct educational meetings/ Audit and feedback	Conduct awareness presentations at PCC	IP, I	Intervention lead presents on MH literacy, stigma reduction, and project activities/updates at each PCC's monthly staffwide meeting	A, I	Prep/ Imp	2x/year	Acceptability, adoption, fidelity, sustainability	Lack of communication between PCC departments about services available; Lack of MH knowledge and MH stigma; Lack of incentive to prioritize MH; Lack of engagement between implementation planners and community stakeholders
Develop educational materials	Base training in real cases	IP	Demonstrate evidence base of IPT-AG and include locally relevant examples of depressed adolescents and treatment in IPT-AG didactic	I	Prep	Once	Acceptability, adoption, fidelity	High valuation of evidence-based interventions; Concern around contextual relevance of a non-locally developed intervention
Provide clinical supervision	Supervise IPT-AG providers	IP	Following didactic training, supervision of 2 IPT-A groups by IPT-AG expert trainer and local IPT-AG expert	I	Prep	Once	Fidelity	Limited confidence in being able to deliver MH services
Change record systems	Create a screening record	IP	Develop paper form for each screener including # adolescents screened and # referred for IPT-AG, collected and reviewed by intervention team lead each week	I	Prep	Once, Cont. Use	Fidelity	Lack of coordination between PCC services and poor referral systems
Develop and organize quality monitoring systems	Meetings between intervention team lead and implementation planners	IP	Intervention team lead reports PCC screening and referral numbers to implementation planners	I	Imp	Weekly	Adoption, fidelity	Lack of coordination between PCC services and poor referral systems
	Continuous communication between implementation planners and team lead	IP	Open communication between implementation planners and intervention team lead to resolve time-sensitive issues	I	Imp	Cont.	Fidelity, penetration, retention	Lack of coordination between PCC services and poor referral systems
	Meetings with implementation planners and intervention team	IP, I	Intervention team lead reports on program fidelity, penetration, and retention and holds open discussion on feedback from adolescents/caregivers and resolving emerging implementation barriers	I	Imp	Monthly	Fidelity, penetration, retention	Lack of coordination between PCC services and poor referral systems
Conduct ongoing training	Conduct refreshment training for screening and IPT-AG providers	IP	Revision of cases and open discussion with providers, IPT-AG expert trainer and local IPT-AG expert	I	Imp	2x/year	Fidelity	Ongoing supervision, monitoring, and technical support after training
**Screening strategies**								
Revise professional roles	Screening by all PCC providers	IP	Screening by general providers (nurses, medicine technicians, counselors) in all departments attending to adolescents	I	Imp	Cont.	Penetration	Specialized health services for adolescents, but with limited personnel, space, privacy
Develop educational materials	Distribute support materials for screening	IP	Post visual materials with screen instructions and scoring algorithm in PCC	I	Imp	Once	Fidelity, penetration	Limited confidence in being able to deliver MH services
–	Use non-stigmatizing language to introduce screen to adolescents	I	Providers use clear, simple, age-appropriate language to describe screen	P	Imp	Cont.	Acceptability, penetration	Low MH literacy and high stigma at the community-level
Change physical structure and equipment	Identify adequate space for screening	I	Intervention team finds or creates quiet, private space	P	Imp	Cont.	Fidelity, penetration	Specialized health services for adolescents, but with limited personnel, space, privacy
**Referral strategies**								
–	Use non-stigmatizing language to give feedback on screen results	I	Providers use simple terms (e.g., sadness) and normalize depression	P	Imp	Cont.	Acceptability, penetration	Low MH literacy and high stigma at the community-level
Revise professional roles	Provide psychoeducation following positive screen	I	Providers describe the importance of treatment and gives overview of IPT-AG	I, P	Imp	Cont.	Penetration	Low MH literacy and high stigma at the community-level
	Bring adolescent with positive screen directly to MH department	I	Providers deliver adolescents along with paper screen in MH providers	I, P	Imp	Cont.	Fidelity, penetration	Lack of coordination between PCC services and poor referral systems
Intervene with patients to promote uptake and adherence	Identify caregiver to participate in IPT-AG sessions with adolescent	I	Providers explain the role of caregivers in IPT-AG and decide with adolescent who is the appropriate person to involve	P	Imp	Cont.	Acceptability, penetration, retention	Involvement of caregivers considered important but challenging to realize
	Call adolescent and/or caregiver on day prior to initial IPT-AG session	I	Provider contacts adolescent and/or caregiver to remind them of upcoming session	P	Imp	Cont.	Penetration	Low MH literacy and high stigma at the community-level; Involvement of caregivers considered important but challenging to realize
**Treatment strategies**								
Revise professional roles	Training of at least 3 providers in each PCC	IP	Inclusion of a MH specialist and 2 non-specialists as IPT-AG providers.	I	Imp.	Cont.	Acceptability, fidelity, sustainability	Frequent provider turnover; Limited confidence in being able to deliver MH services
	MH specialist and general provider deliver groups together	IP	Groups led by MH specialist and a non-specialist together for first 6 months.	I	Imp.	6 mo.	Acceptability, fidelity, sustainability	Frequent provider turnover; Limited confidence in being able to deliver MH services
Intervene to promote uptake and adherence	Morning, afternoon, and Saturday groups offered	I	Work with adolescents and providers to identify best time for them to participate in sessions	P	Imp.	Cont.	Acceptability, retention	Need for multiple, lengthy sessions
Promote adaptability	Weekly group sessions	I	Hold IPT-AG sessions weekly	P	Imp.	Cont.	Acceptability, fidelity, retention	^**^Determined feasible and preferable in workshops
Change physical structure and equipment	Identify adequate space for sessions	I	Intervention team finds or creates quiet, private, open space	P	Imp.	Cont.	Acceptability, retention	Specialized health services for adolescents, but with limited personnel, space, privacy
Promote adaptability	Age-appropriate age composition	I	Composition of groups with adolescents 12–14 and 15–19	P	Imp.	Cont.	Acceptability, retention	^**^Determined as appropriate age groups in workshops
Intervene to promote uptake and adherence	Educate adolescent about IPT-AG	I	Educate adolescent on IPT-AG treatment objectives, duration, content	P	Imp.	Cont.	Retention	Need for multiple, lengthy sessions
	Call adolescent and/or caregiver on day prior to each session	I	Provider contacts adolescent and/or caregiver to remind them of upcoming session	P	Imp.	Cont.	Retention	Need for multiple, lengthy sessions
	Include caregivers remotely when they are unable to join session at PCC	I	Use phone or online platforms to include caregivers in sessions	P	Imp.	Cont.	Acceptability, retention	Involvement of caregivers considered important but challenging to realize

*Temp, Temporality; IP, Implementation Planners; A, Adopters; I, Implementers; C, Community; P, Patien; Prep, Preparation Phase; Imp, Implementation Phase; Cont., continuous; PCC, Primary Care Clinic; IPT-AG, Group Interpersonal Therapy for Adolescents; MH, Mental Health;*

* *Justification based on corresponding implementation determinant targeted by strategy*,

** *Justification based on stakeholder workshops and not qualitative formative assessment*.

### Implementation Materials and Evaluation of Implementation Outcomes

**Figure 1:**
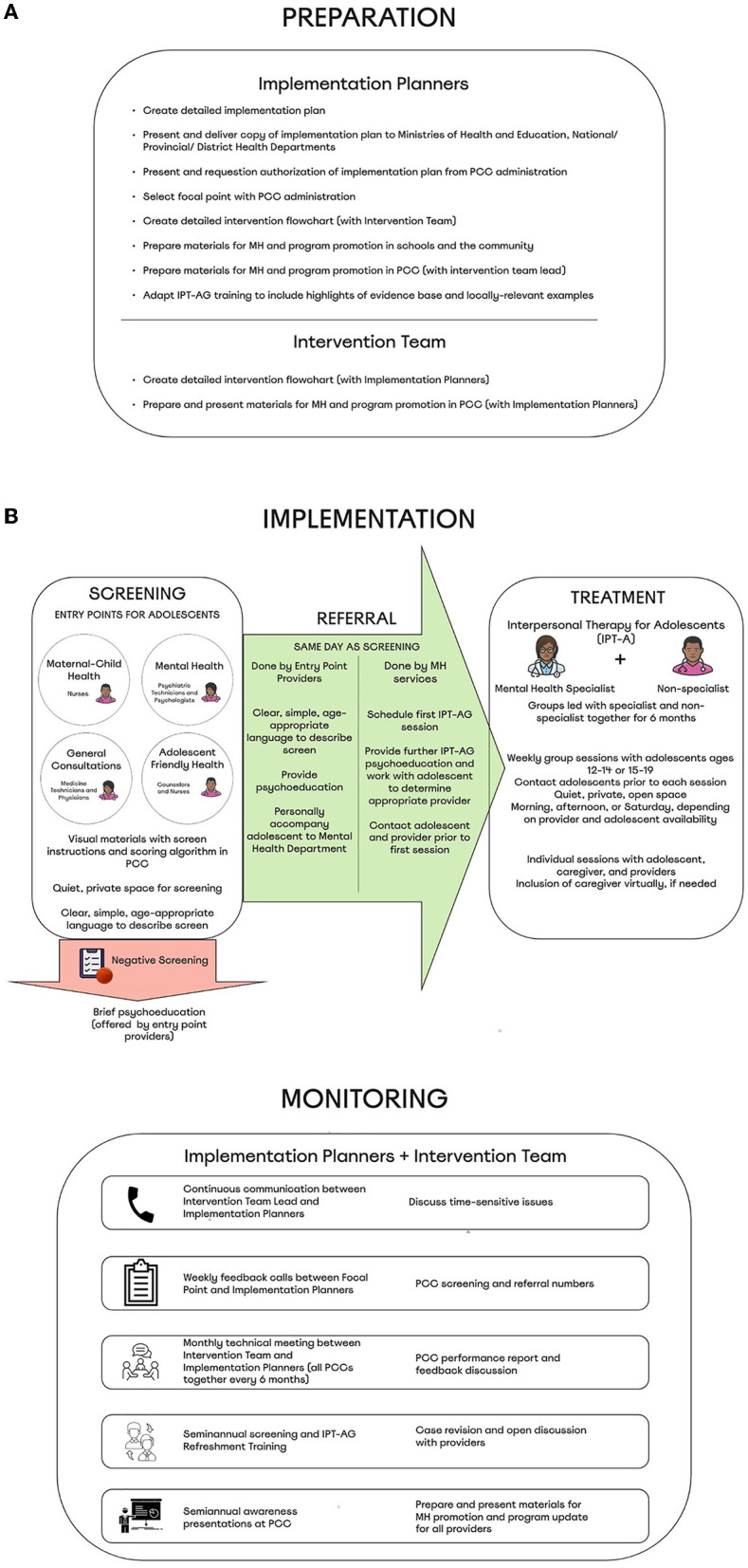
**(A,B)** Implementation plan for adolescent depression services integrated within Mozambican primary care.

We will examine the patient and implementation outcomes associated with our finalized implementation plan (Figure 1) in a hybrid type II cluster randomized trial in PCC of Maputo, Mozambique. Protocols and materials for preparation and implementation of the trial are guided by strategies included in the final implementation plan. Specifically, we are currently developing a more detailed implementation plan that includes objectives, roles, activities, timeline, budget, and expected outcomes of the project. We are also working with the Ministries of Health and Education to develop materials (e.g., presentations, flyers) for a mental health awareness campaign to be delivered in schools and communities. Moreover, we will work with intervention implementers to create a presentation to promote general mental health awareness as well as project-specific activities in each of the participating PCC. We will also work with intervention implementers to design the detailed intervention flowchart for each PCC. Finally, we are adapting IPT-AG training materials to highlight the evidence base, include guidance on choosing an appropriate caregiver with the adolescent, and incorporate locally-relevant examples; creating a screening record to be used for quality control; and developing visual guides for conducting and scoring screening measures that will be posted in all PCC departments. Results of this pilot trial will be used to inform any modifications needed to the present implementation plan, for example additional strategies needed to promote treatment fidelity or to manage and promote retention among adolescents between initial screening and IPT-A groups.

## Discussion

Despite the enormous mental health treatment gap, there is still very limited data on effective strategies for implementing mental services in LMIC, especially with regard to adolescent mental health services. The systematic selection of implementation strategies is critical to the success of a program as well as our understanding of the effectiveness of different implementation strategies across programs (29). We present here, to our knowledge, the first application of Implementation Mapping to develop an implementation plan for LMIC settings. We demonstrate that using a blend of in-person and virtual approaches for Implementation Mapping activities can facilitate international implementation planning partnerships and the engagement of multilevel stakeholders. Additionally, we identify a number of unique implementation determinants and strategies important for adolescent mental health care integration in PCC that have not previously been noted for implementation of adult mental health care in LMIC. In the coming years, the implementation plan developed here will be evaluated for delivering adolescent depression services in Mozambican primary care and may serve as a model for other low-resource settings.

The use of Implementation Mapping provided a systematic process employing theory, evidence, and stakeholder engagement to develop our implementation plan (19). Incorporating both virtual and in-person approaches provided the flexibility necessary for international work while maintaining fidelity to this structured process. One of the main ways that virtual tools were employed was for implementation planner activities (e.g., remote meetings, online qualitative data analysis with Dedoose, logic models built in Miro). While adjustment to use of these tools required additional time, they permitted the consistent involvement of local partners, which was critical to the veracity and contextual relevance of data. For example, all qualitative data was analyzed in Portuguese, rather than translating to English for analysis then back-translating for presentation at workshops, limiting data loss across activities. Virtual tools were also used to rapidly adapt during COVID-19 related restrictions on in-person activities (e.g., qualitative interviews over Zoom), highlighting their importance in an agile research process. Still, while virtual tools supported engagement that would otherwise not be possible, in-person activities continued to be invaluable to the process. Specifically, in-person workshops promoted communication and engagement between stakeholders ranging from junior PCC providers to high-ranking Ministry officials, which, in turn, resulted in the selection and specification of strategies informed by diverse perspectives, an integral component to effective implementation as well as future scale-up and sustainability of the program (27).

A recent systematic review of determinants to implementing adult mental health services in LMIC primary care found a number of common barriers and facilitators (8). Across CFIR levels, our findings were consistent with those previously demonstrated. For example, research from multiple other LMIC have similarly demonstrated the need for lengthy visits (30, 31), low mental health literacy and high levels of stigma in communities (30, 32–36), and poor communication and referral systems in PCC (37–39) as barriers as well as provider perception that mental health care integration is important as a facilitator (31, 40–42) to mental health service integration. Unique in our study, however, are determinants which may serve as important targets of implementation strategies for interventions addressing adolescent mental health in this and other settings. For example, involvement of caregivers was considered very important but challenging to realize. We therefore included strategies to promote the inclusion of a caregiver in a way that is acceptable to both the adolescent (e.g., providers working with adolescents to select the appropriate caregiver) and the caregiver themself (e.g., reminding caregivers of the session the day before and creating options for joining remotely if caregivers are unable to travel to the PCC). As a 2020 systematic review on implementation of depression interventions in LMIC did not identify a single study focused on implementation strategies for youth (child or adolescent) populations (9), further research on adolescent-specific implementation determinants and effective implementation strategies to target these determinants is urgently needed.

To further ground our study in implementation science, in addition to using Implementation Mapping to guide our process, we employed specific implementation frameworks in our selection of implementation outcomes (i.e., Proctor's Implementation Outcome Framework) (21), investigation of implementation determinants (i.e., CFIR) (22), selection of potential strategies (i.e. ERIC) (25), and project synthesis (i.e., Implementation Logic Models) (24). While use of these frameworks promoted the rigor and specification of our process, we encountered a number of challenges in their application. For one, while the CFIR domains were relevant to the present study, the specific constructs within each were not as obvious in their application to the context and project, causing us to shift from using a best-fit framework approach to an open-coding approach for qualitative analysis. Our experience is consistent with a systematic review that demonstrated a number of CFIR constructs to be considered incompatible or irrelevant by investigators using them in LMIC settings and suggested adaptations to the CFIR be made for use in these contexts (43). Moreover, while the potential strategies we selected were generated by reviewing the ERIC strategies and adapting them to the context, when mapping our finalized strategies back onto the ERIC during strategy specification, we found that individuals strategies at times appeared to fit into several different ERIC strategies. For example, we matched our strategy “Create a screening record” as the ERIC strategy *change record systems*, but it also could have mapped to *develop and implement tools for quality monitoring*. We therefore chose to select ERIC strategy matches by which we felt best captured our strategy's objective (i.e., the justification and implementation outcome targeted). Our experience supports a recent call to increase focus on the mechanisms of implementation strategies (29) rather than the strategies themselves, which are less readily compared across studies. Finally, in preparing the logic models for workshops, we determined that simplifying the models, like changing the names of CFIR domains to project-specific counterpart (e.g., PCC instead of inner setting), would allow stakeholders to more easily understand and interact with them. We share these experiences not to undercut the importance of using implementation frameworks in LMIC settings, but rather to highlight the need to adapt to the context and prioritize program goals in their application.

The results presented here should be considered in light of the following limitations. For one, qualitative implementation determinant data collection and implementation strategy selection workshops occurred in one province. While we included PCC providers from urban and periurban regions as well as policymakers and NGO representatives that serve multiple provinces, adaptations may be needed to the implementation plan to meet the needs and assets of other Mozambican provinces where care-seeking and cultural norms, such as gender roles, may differ and which have more limited PCC staff and mental health providers. Additionally, owing to the COVID-19 related restrictions on in-person activities, we were unable to include community members (e.g., adolescents, caregivers, traditional healers) in our exploration of implementation determinants. Future research with community members should be explored to understand additional determinants (e.g., stigma, health beliefs) and strategies to further improve contextual relevance of the implementation plan. Finally, the vast majority of implementation strategies proposed were ranked as high priority (both feasible and important). In this project, we were able to include all high priority strategies in the implementation plan; however, for other projects in which it is not possible to include a large number of strategies within the implementation plan, it may be necessary to use a different prioritization methodology. We grouped participant feedback from the 2 × 2 table into four categories because, when we asked workshop participants to rank strategies within each quadrant, they informed us that they generally believed the strategies within each quadrant to be equally important/feasible, unless they had clearly placed the strategy toward the middle axes. In other projects, it may be necessary to better familiarize participants with this type of ranking system and/or require participants to rank strategies so that none are given equal priority.

Despite these limitations, we believe this study provides important contributions to the literature. To our knowledge, this is one of the first studies to systematically develop a strategy for implementation of adolescent mental health services and the first to apply Implementation Mapping in LMIC. Findings from this study will inform future scale-up of integrated adolescent mental health services in Mozambique and may serve as a model for efforts in other LMIC. Additionally, the use of virtual tools to facilitate an international research-policy partnership and implementation activities demonstrates a flexible application of Implementation Mapping that can promote diverse stakeholder engagement.

## Data Availability Statement

The raw data supporting the conclusions of this article will be made available by the authors, without undue reservation.

## Ethics Statement

All study materials and procedures were approved by the New York State Psychiatric Institute Institutional Review Board and the National Committee for Bioethics in Health of Mozambique. The patients/participants provided their written informed consent to participate in this study.

## Author Contributions

KL conceived of and designed the study with support from PS, CD, RB, and MW. PS, SA, CB, MF, BK, TR, and AJ managed data collection. KL, SA, CB, MF, BK, TR, and AJ contributed to data analysis. KL wrote the initial draft of the manuscript. All authors contributed to manuscript editing and have approved the final draft for publication.

## Funding

This project was supported by the National Institute of Mental Health (K01MH120258).

## Conflict of Interest

The authors declare that the research was conducted in the absence of any commercial or financial relationships that could be construed as a potential conflict of interest.

## Publisher's Note

All claims expressed in this article are solely those of the authors and do not necessarily represent those of their affiliated organizations, or those of the publisher, the editors and the reviewers. Any product that may be evaluated in this article, or claim that may be made by its manufacturer, is not guaranteed or endorsed by the publisher.
