# Supplementary material for: Leveraging Stakeholder Engagement and Virtual Environments to Develop a Strategy for Implementation of Adolescent Depression Services Integrated Within Primary Care Clinics of Mozambique
Source: Front Public Health. 2022 May 26;10:876062. doi: 10.3389/fpubh.2022.876062 (PMC9178075; doi:10.3389/fpubh.2022.876062)
Supplement: Supplementary file 1 [file Table_1.DOCX]

Supplementary Material

Supplementary Figure 1. Flow Diagram of Implementation Mapping Approach

Supplemental Figure 2. Miro Diagram of Simplified Implementation Research Logic Models


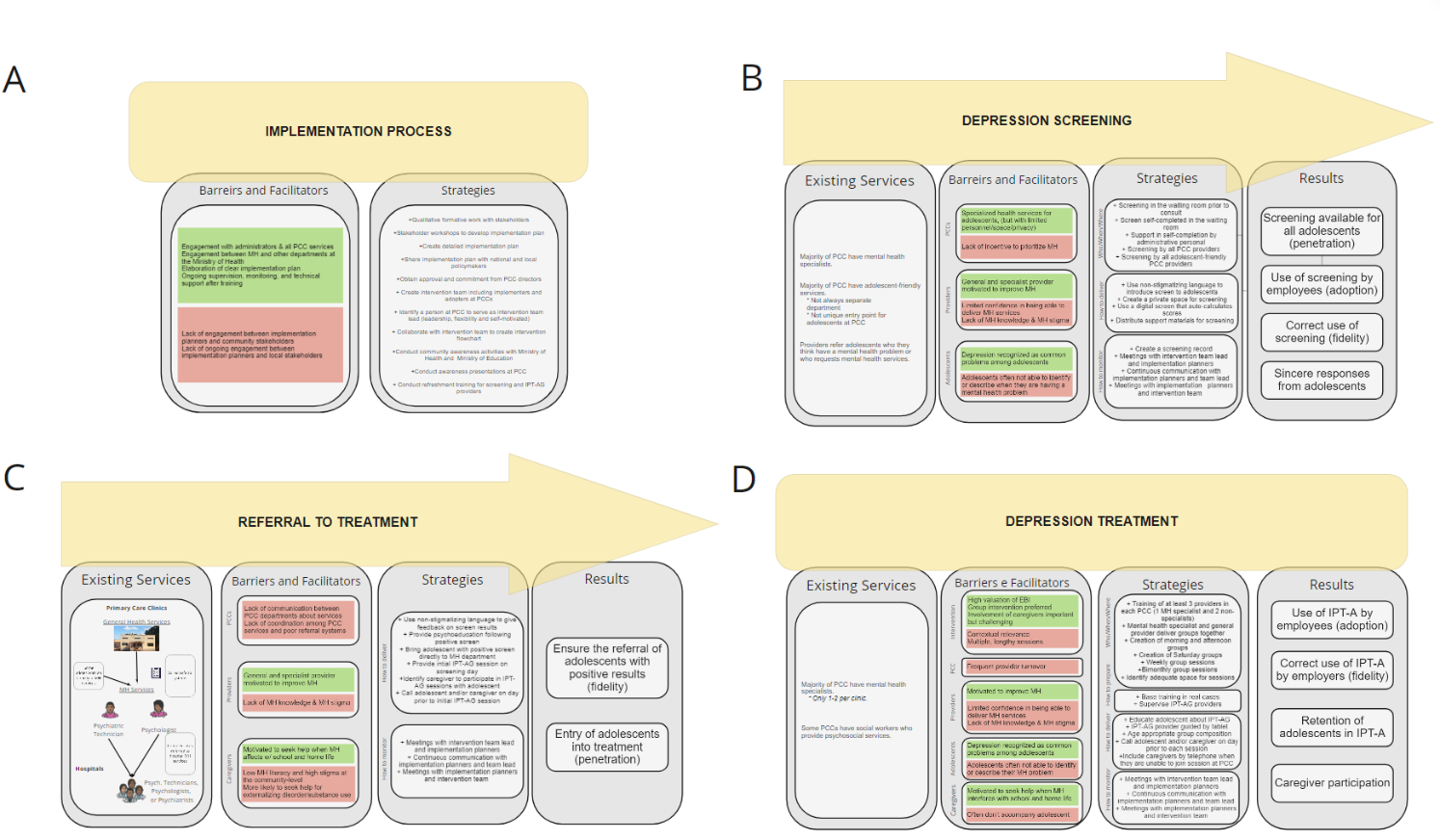


Logic Models for Implementation Process (A), Depression Screening (B), Referral (C), and Treatment. Green boxes include facilitators and red boxes include barriers identified in qualitative implementation determinants investigation.

Supplemental Figure 3. Example Miro Diagram of Implementation Strategy Ranking


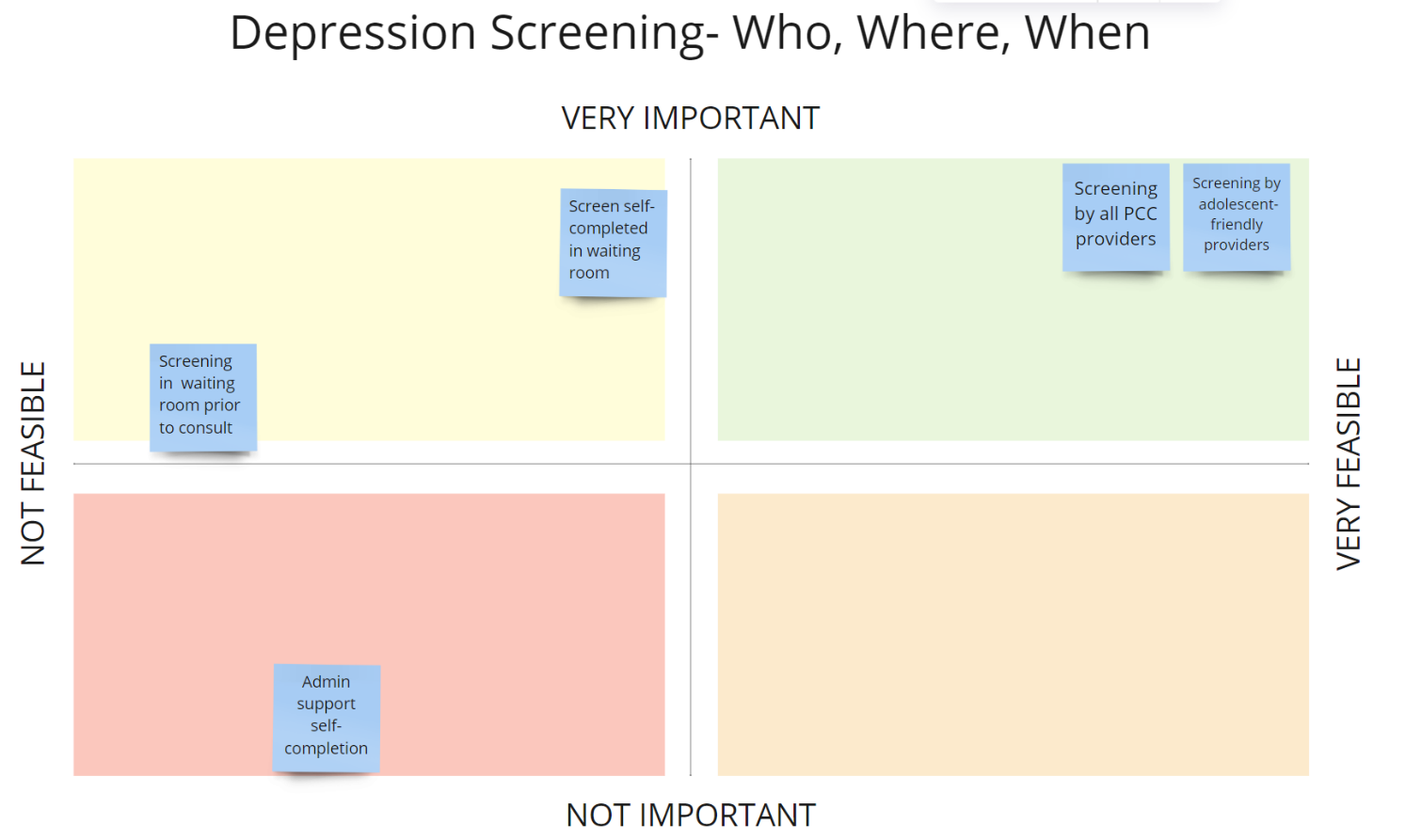


2x2 table showing stakeholder prioritization of implementation strategies for delivery of depression screening.
